# Alteration of *POLDIP3* Splicing Associated with Loss of Function of TDP-43 in Tissues Affected with ALS

**DOI:** 10.1371/journal.pone.0043120

**Published:** 2012-08-10

**Authors:** Atsushi Shiga, Tomohiko Ishihara, Akinori Miyashita, Misaki Kuwabara, Taisuke Kato, Norihiro Watanabe, Akie Yamahira, Chigusa Kondo, Akio Yokoseki, Masuhiro Takahashi, Ryozo Kuwano, Akiyoshi Kakita, Masatoyo Nishizawa, Hitoshi Takahashi, Osamu Onodera

**Affiliations:** 1 Department of Pathology, Brain Research Institute, Niigata University, Niigata, Japan; 2 Department of Neurology, Brain Research Institute, Niigata University, Niigata, Japan; 3 Department of Molecular Genetics, Genome Science Branch, Center for Bioresource-Based Researches, Brain Research Institute, Niigata University, Niigata, Japan; 4 Laboratory of Hematology and Oncology, Graduate School of Health Sciences, Niigata University, Niigata, Japan; 5 Department of Pathological Neuroscience, Resource Branch for Brain Disease Research, Brain Research Institute, Niigata University, Niigata, Japan; 6 Department of Molecular Neuroscience, Resource Branch for Brain Disease Research, Brain Research Institute, Niigata University, Niigata, Japan; National Center of Neurology and Psychiatry, Japan

## Abstract

Amyotrophic lateral sclerosis (ALS) is an adult-onset neurodegenerative disease caused by selective loss of motor neurons. In the ALS motor neurons, TAR DNA-binding protein of 43 kDa (TDP-43) is dislocated from the nucleus to cytoplasm and forms inclusions, suggesting that loss of a nuclear function of TDP-43 may underlie the pathogenesis of ALS. TDP-43 functions in RNA metabolism include regulation of transcription, mRNA stability, and alternative splicing of pre-mRNA. However, a function of TDP-43 in tissue affected with ALS has not been elucidated. We sought to identify the molecular indicators reflecting on a TDP-43 function. Using exon array analysis, we observed a remarkable alteration of splicing in the polymerase delta interacting protein 3 (*POLDIP3*) as a result of the depletion of TDP-43 expression in two types of cultured cells. In the cells treated with TDP-43 siRNA, wild-type *POLDIP3* (variant-1) decreased and *POLDIP3* lacking exon 3 (variant-2) increased. The RNA binding ability of TDP-43 was necessary for inclusion of *POLDIP3* exon 3. Moreover, we found an increment of *POLDIP3* variant-2 mRNA in motor cortex, spinal cord and spinal motor neurons collected by laser capture microdissection with ALS. Our results suggest a loss of TDP-43 function in tissues affected with ALS, supporting the hypothesis that a loss of function of TDP-43 underlies the pathogenesis of ALS.

## Introduction

Amyotrophic lateral sclerosis (ALS) is an adult-onset neurodegenerative disease accompanied by loss of motor neurons. A pathological hallmark of ALS is cytoplasmic inclusions in neurons and glia consisting of TAR DNA-binding protein of 43 kDa (TDP-43) [1], [2], [3]. Although TDP-43-positive inclusions have been identified in several neurodegenerative disorders, the discovery of mutations in the *TARDBP* gene in patients with familial and sporadic ALS (OMIM 612069; ALS10) indicates that an alteration of TDP-43 causes selective motor neuron degeneration [4], [5]. The similarity between the pathological findings in ALS10 and sporadic ALS indicate that the alteration of TDP-43 may play an important role in the pathogenesis of sporadic ALS [3], [6]. However, the way in which the molecular mechanism of this alteration of TDP-43 causes ALS is still obscure.

TDP-43 is a ubiquitously expressed nuclear protein and the clearance of nuclear TDP-43 in the affected neurons and glia is another pathological hallmark of ALS, suggesting that a loss of function of TDP-43 may underlie the pathogenesis of the ALS [2], [3], [7]. TDP-43 has two RNA recognition motifs (RRM1 and RRM2) and has been speculated as a heterogeneous nuclear ribonucleoprotein (hnRNP) [8], [9]. TDP-43 binds to hnRNPs and functions in RNA metabolism through regulation of transcription, mRNA stability, and alternative splicing of pre-mRNA [10], [11]. TDP-43 takes an important role for cell proliferation, neurite outgrowth and neuronal cell viability [12], [13]. In animal models, several lines of evidence have revealed that TDP-43 is essential for normal embryogenesis, ES survival and takes an important role for motor neuron function [14], [15], [16], [17]. However, a function of TDP-43 in the human tissues has not been well evaluated.

Controversy centers on whether a loss of TDP-43 function or an adverse effect of inclusions of TDP-43 results in motor neuron death in ALS [7]. The disappearance of nuclear TDP-43 in the affected motor neurons with TDP-43 inclusions supports the hypothesis that a loss of TDP-43 function may result in the motor neuron death in ALS [1], [2], [18]. However, TDP-43 function in tissues affected with ALS has not been evaluated. To explore the possibility that a loss of TDP-43 function may result in the motor neuron death in ALS, a function of TDP-43 in tissues affected with ALS should be elucidated. In this report, we first attempted to identify the molecular indicators of a function of TDP-43 in human cells, with special attention to pre-mRNA splicing. Then we evaluated a function of TDP-43 in the affected tissues with ALS by using the identified molecular indicator, splicing variants of *POLDIP3.*

## Results

### Comprehensive screening of TDP-43 functions in pre-mRNA splicing

**Figure 1:**
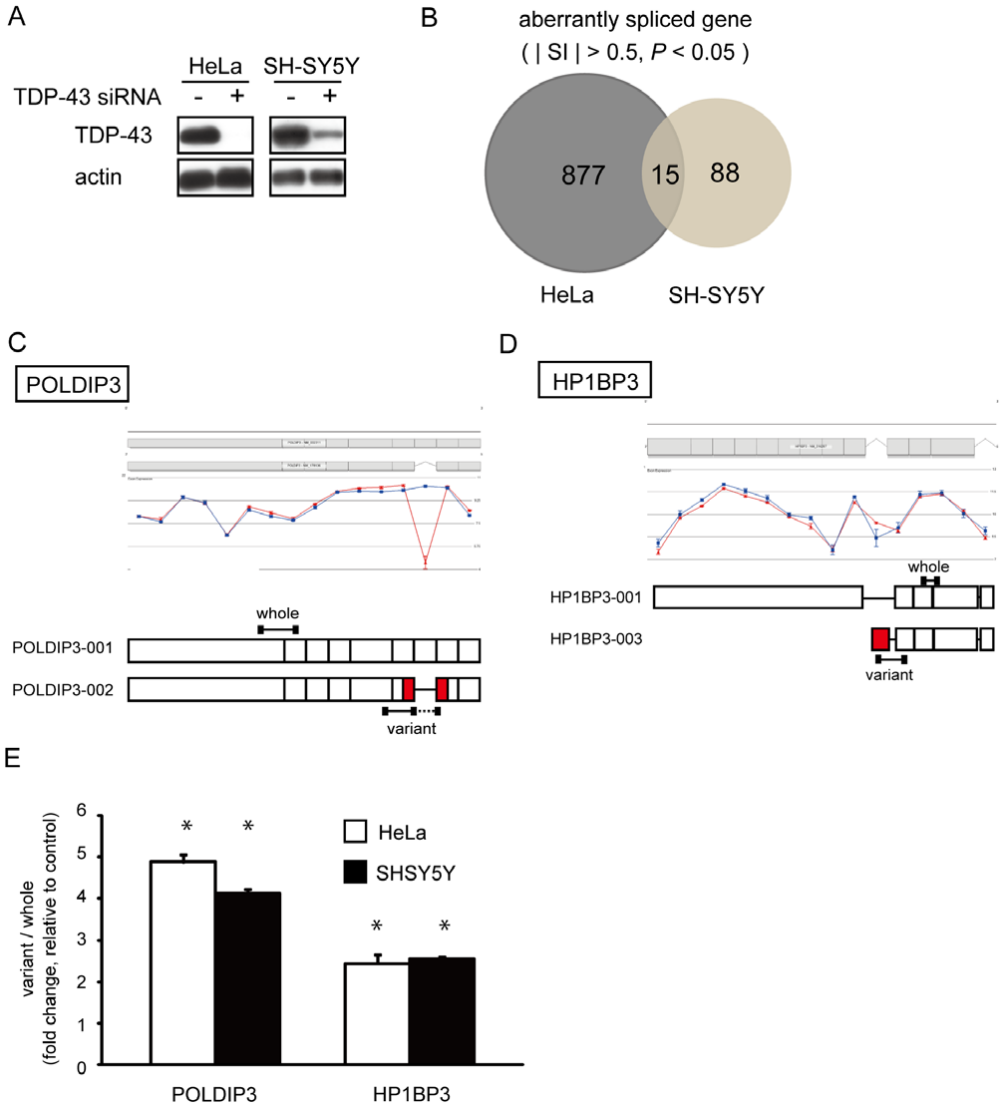
Genes with altered splicing by depletion of TDP-43 in culture cells. (**A**) Western blot analysis of TDP-43 in HeLa and SH-SY5Y cells with or without TDP-43 siRNA. TDP-43 and actin were shown by immunoblotting with anti-TDP-43 (top) and anti-actin (bottom) antibody, respectively. (**B**) Microarray analyses were performed in control (transfected with siControl non-targeting pool) and TDP-43 knockdown HeLa and SH-SY5Y cells in triplicate. Venn diagram shows overlapping of altered splicing genes between HeLa and SH-SY5Y cells. Only 15 genes overlapped between them. From the array data, we calculated the *P* value and the splicing index (|SI|). Both *P*<0.05 and |SI|>0.5 were used as thresholds to identify the genes with altered splicing. (**C**) Exon structure diagram of the main isoform (top) and candidate variant (bottom) for *POLDIP3* and *HP1BP3.* From the exon array result, we selected the candidate splicing variant that was induced by the depletion of TDP-43. The name of each transcript is labeled on the side of the exon structure. The red box indicates the exons that are expected to be altered by TDP-43 depletion. The black bars indicate the position of the primers we used in this experiment. The gene views show the expression of exons as determined by analyzing the results of exon array in HeLa cells using Genespring GX. The expression levels are shown on a log2 scale; the error bars show standard errors of means. TDP-43 siRNA, red circle; control siRNA, blue square. (**D**) qRT-PCR analysis revealed that the splicing alteration of *POLDIP3* and *HP1BP3* gene were validated in both HeLa and SH-SY5Y cells. *RPLP1* and *RPS18* were used as reference genes. Data represent the mean with standard error from three independent experiments. Asterisk indicates significant difference (*P<0.01, Student *t* test).

For investigating the function of TDP-43 in several tissues, ideal molecular indicators are the genes that are ubiquitously expressed and influenced by the depletion of TDP-43. TDP-43 has been reported to bind the intronic or noncoding sequences, which are markedly different between species, and affect pre-mRNA splicing [19], [20]. Therefore we attempted to identify the genes which are influenced by TDP-43 by comparing the results of two differential human cells, HeLa and SH-SY5Y. siRNA treatment completely suppressed the expression of TDP-43 in HeLa cells, in contrast to <30% in SH-SY5Y cells compared to control siRNA (Fig. 1A). We obtained the cDNAs from mRNA of these cells and applied them to GeneChip Human Exon 1.0 ST Arrays, subsequently identifying genes that are significantly altered in their splicing and amount of expression. We found 892 genes in HeLa cells and 103 genes in SH-SY5Y cells that altered the pattern of splicing under the depletion of TDP-43 (Fig. 1B). In contrast, we found 123 genes, 98 genes upregulated and 25 genes down regulated, in HeLa cells and 10 genes, 3 genes upregulated and 7 genes downregulated, in SH-SY5Y cells in which the amounts of mRNA were significantly altered more than 2-fold by TDP-43 siRNA compared to control siRNA.

### *POLDIP3* exon 3 is excluded by depletion of TDP-43

Among the genes that altered their splicing, we found that only 15 genes overlapped between both cell lines (Fig. 1B and Table 1). By comparing the results of the exon array to the exon structure information in the Ensemble (http://asia.ensembl.org/index.html), we found candidate exon cassettes in six genes (polymerase delta interacting protein 3 [*POLDIP3*]; methyl malonic aciduria cblB type [*MMAB*]; heterochromatin protein 1, binding protein 3 [*HP1BP3*]; glutaminase [*GLS*]; stimulated by retinoic acid gene 6 homolog [*STRA6*]; and dolichyl-phosphate mannosyltransferase polypeptide 2, regulatory subunit [*DPM2*]) for further experiments to validate the exon array results (Fig. 1B and Table 1). Quantitative reverse-transcriptase polymerase chain reaction (qRT-PCR) analysis using primers specific for splicing variants revealed that only the *POLDIP3* and *HP1BP3* genes showed altered splicing consistent with the results of an exon array in both HeLa cells and in SH-SY5Y cells (Fig. 1C and D, Fig. S1A and B).

**Table 1:** Aberrantly spliced genes overlapping between HeLa and SH-SY5Y cells.

Gene Symbol	Gene Name	Splicing Index (HeLa)	Splicing Index (SH-SY5Y)	Splicing ANOVA Corrected p-value (HeLa)	Splicing ANOVA Corrected p-value (SH-SY5Y)	RefSeq
*POLDIP3*	polymerase (DNA-directed), delta interacting protein 3	−5.061	−1.967	3.57E-36	8.85E-09	NM_032311
*SMC1A*	structural maintenance of chromosomes 1A	−1.664	−1.552	8.60E-28	2.99E-12	NM_006306
*MMAB*	methylmalonic aciduria (cobalamin deficiency) cblB type	−1.177	−1.149	0.00134	7.85E-04	NM_052845
*HP1BP3*	heterochromatin protein 1, binding protein 3	1.132	1.116	0.00906	0.03201	NM_016287
*GLS*	glutaminase	−1.062	−1.037	4.12E-09	3.42E-07	NM_014905
*STRA6*	stimulated by retinoic acid gene 6 homolog (mouse)	1.053	1.077	2.91E-06	4.10E-05	NM_022369
*RNFT2*	ring finger protein, transmembrane 2	1.028	−0.692	2.40E-06	0.00511	NM_001109903
*DPM2*	dolichyl-phosphate mannosyltransferase polypeptide 2, regulatory subunit	0.993	0.929	0.00204	0.02473	NM_003863
*JAGN1*	jagunal homolog 1 (Drosophila)	0.941	0.991	6.54E-04	2.25E-04	NM_032492
*BPNT1*	3′(2′), 5′-bisphosphate nucleotidase 1	0.933	1.108	0.02682	7.85E-04	NM_006085
*CDCA3*	cell division cycle associated 3	0.931	0.745	0.03150	0.00220	NM_031299
*LOC729927*	similar to 40S ribosomal protein S20	−0.827	0.913	0.00721	0.03092	-
*OLFM1*	olfactomedin 1	0.783	0.552	1.62E-04	0.00220	NM_014279
*CPNE1*	copine I	0.635	0.573	0.01643	0.04548	NM_152927
*VEGFA*	vascular endothelial growth factor A	0.614	0.627	0.01974	0.01099	NM_001025366

**Figure 2:**
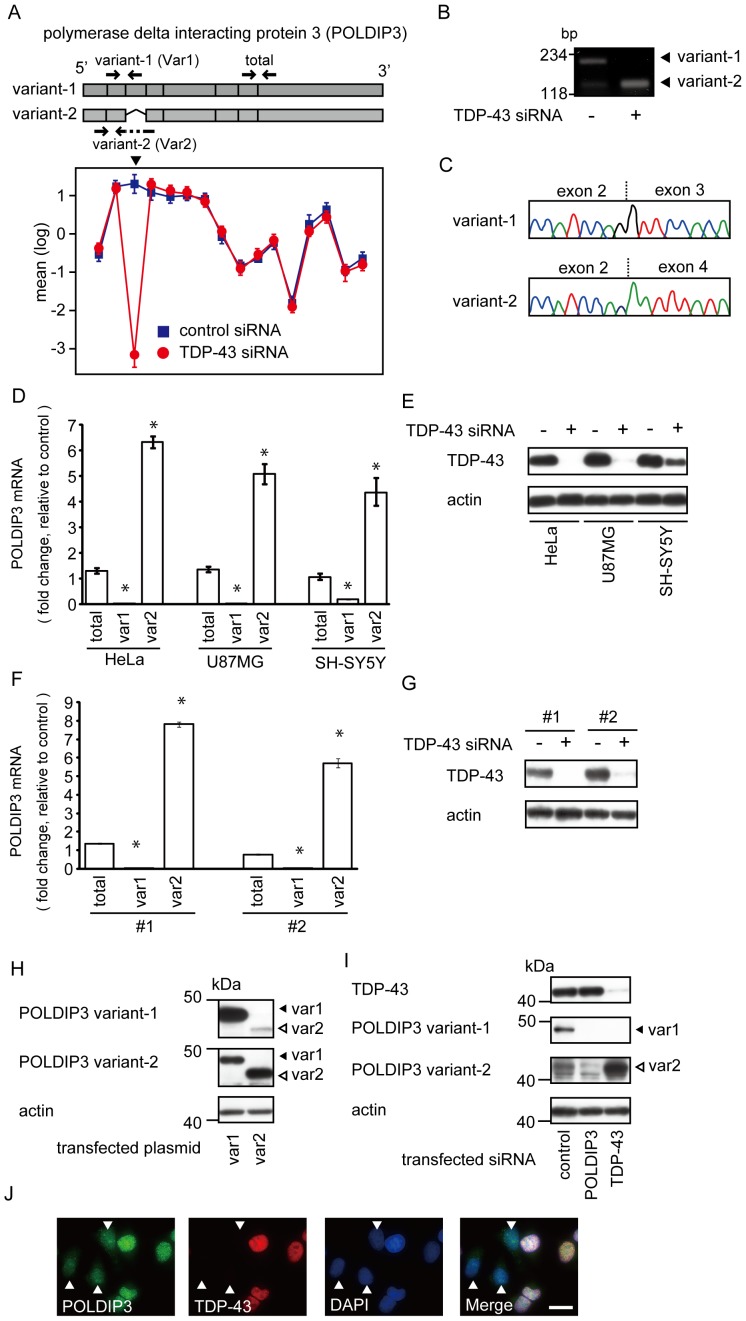
Depletion of TDP-43 mRNA causes aberrant splicing of *POLDIP3* mRNA. (**A**) Alteration of the splicing patterns of *POLDIP3* exon 3. The exon structure of the *POLDIP3* variant-1 (including exon 3) and variant-2 (lacking exon 3) (upper) are shown. Boxes indicate each exon. Arrows indicates the position of primer for amplification of each splicing variant (variant-1 or variant-2) and both *POLDIP3* variants (total). These primers were used in Fig. 2D and F. The gene views show the expression of exons as determined by analyzing the results of the exon array in HeLa cells using Genespring GX. The expression levels are shown on a log2 scale; the error bars show standard errors of means. TDP-43 siRNA, red circle; control siRNA, blue square. Arrowhead shows the probe on *POLDIP3* exon 3. (**B**) The results of an RT-PCR assay with the use of primers spanning *POLDIP3* exon 3 from control or TDP-43 deleted cells (indicate as TDP-43 siRNA +). (**C**) The electropherograms show that variant-1 includes exon 3, whereas variant-2 excludes exon 3. (**D** and **F**) *POLDIP3* mRNA levels in culture cells transfected with TDP-43 siRNA as a fold change relative to those of cells transfected with control siRNA. The amounts of variant-1 (var1), variant-2 (var2), or both (total) *POLDIP3* mRNA were quantified using primers indicated with arrows in Fig. 2A. *RPLP1* and *RPS18* were used as reference genes. Data represent the mean with standard error from three independent experiments. Asterisk indicates significant difference (*P<0.01, Student *t* test). (**D**) Three different cultured cell lines were transfected with TDP-43 siRNA. (**F**) HeLa cells transfected with two additional TDP-43 siRNAs (#1 and #2). (**E** and **G**) Expression of TDP-43 in analyzed cells. TDP-43 was effectively depleted in cells transfected with TDP-43 siRNA. (**H**) Evaluation of anti-POLDIP3 antibodies for each splicing variant of POLDIP3. HEK293T cells were transfected with a POLDIP3 variant-1 or POLDIP3 variant-2 expression vector. POLDIP3 is shown by immunoblotting with the use of the anti-POLDIP3 variant-1 antibody (var1) or anti-POLDIP3 variant-2 antibody (var2). Note that variant-1 antibody reacts predominantly with POLDIP3 variant-1 (upper panel), and variant-2 antibody reacts predominantly with POLDIP3 variant-2 (middle panel). Anti-actin immunoblotting served as a loading control. (**I**) Depletion of TDP-43 results in a decrement of POLDIP3 variant-1 and increment of variant-2. Cell lysates from the HEK293T cells transfected with POLDIP3 or TDP-43 siRNA were subjected to immunoblotting for TDP-43, POLDIP3 variant-1, and POLDIP3 variant-2. Anti-actin immunoblotting served as a loading control. (**J**) Immunohistochemical analysis of POLDIP3 variant-1. HeLa cells were transfected with TDP-43 siRNA, followed by immunostaining with both anti-POLDIP3 variant-1 antibody and anti-TDP-43 antibody. Arrowheads indicate TDP-43-depressed cells. Scale bar, 20 µm.

We were interested in *POLDIP3* because *POLDIP3* showed the highest fold change by qRT-PCR in both cell lines (Fig. 1E). The mean gene-normalized probe-set signals for *POLDIP3* revealed the lack of exon 3 as a result of the depletion of TDP-43 (Fig. 2A). RT-PCR analysis using primers spanning exon 3 revealed that the cells treated with TDP-43 siRNA showed only one product; subsequent sequence analysis showed that it was from the transcripts excluding *POLDIP3* exon 3 (Fig. 2B and C). We designated the *POLDIP3* gene lacking exon 3 as *POLDIP3* variant-2 and wild-type as variant-1.

We also quantified the amounts of *POLDIP3* splicing variants mRNA in various human cultured cells, HeLa, SH-SY5Y, and U87-MG, by qRT-PCR using primers that specifically amplified each splice variant. Under treatment with TDP-43 siRNA, the total amount of *POLDIP3* in these cells was not significantly altered, whereas variant-1 was significantly reduced to <5% and variant-2 was increased to more than 4 to 6 times compared to the control (Fig. 2D and E). Similar results were obtained using two other mixtures of TDP-43 siRNAs (Fig. 2F and G).

We then performed Western blot analysis using anti-POLDIP3 antibodies that predominantly react with POLDIP3 variant-1 or POLDIP3 variant-2 (Fig. 2H). In the cells treated with TDP-43 siRNA, POLDIP3 variant-1 decreased and variant-2 increased remarkably (Fig. 2I). Next, we performed immunohistochemical analysis using POLDIP3 variant-1 antibody. This antibody clearly showed immunoreactivity for POLDIP3 variant-1 in the nucleus in cells with TDP-43 as previously reported [21] (Fig. 2J). However, the immunoreactivity for nuclear POLDIP3 variant-1 was decreased in the cells with decreased TDP-43 expression (Fig. 2J).

### RNA binding ability of TDP-43 is necessary for including *POLDIP3* exon 3

**Figure 3:**
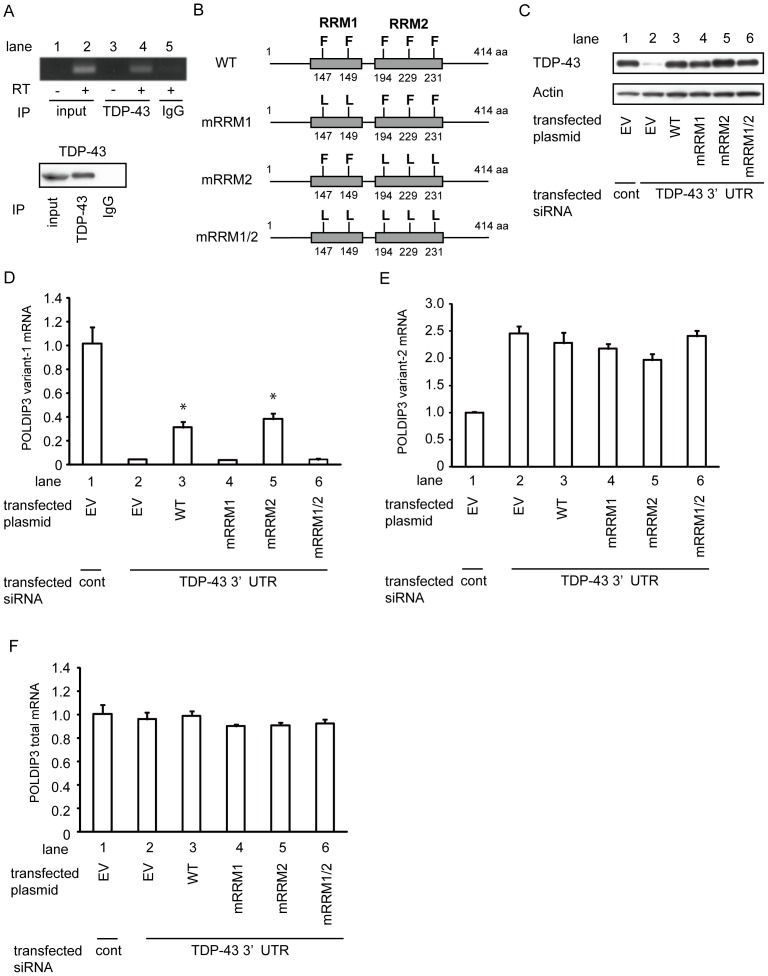
TDP-43 RRM1 domain is necessary for including *POLDIP3* exon 3. (**A**) TDP-43 binds to *POLDIP3* mRNA. Whole cell lysate from HeLa cells was immunoprecipitated with anti-TDP-43 or control rabbit IgG, subjected to the isolation of RNA and RT-PCR analysis using primers located in exon 5 of *POLDIP3*. (**B–F**) Analysis of *POLDIP3* splicing by TDP-43 with mutated in RRM domains. (**B**) Schematic diagram of the constructs for wild-type (WT) and mutated TDP-43 expression plasmid (mRRM1, mRRM2, and mRRM1/2). Phe (F) to Leu (L) alterations were introduced in RRM1, RRM2, or both motifs of the TDP-43. Boxes indicate RRM motif. Numbers indicate the location of substituted amino acids. (**C**) TDP-43 depletion caused by siRNA targeting 3′-UTR of *TDP-43* (TDP-43 3′-UTR siRNA) was rescued by ectopic expression of WT and mutated TDP-43 in HEK293T cells. Mutated TDP-43s were similarly expressed. HEK293T cells were transfected with control (lane 1) or TDP-43 siRNA targeting for 3′-UTR region (lanes 2–6), followed by transfection with empty vector (EV) or various TDP-43 expression plasmids. TDP-43 expression was assessed by probing with anti-TDP-43 antibody. Anti-actin immunoblotting served as a loading control. (**D**–**F**) *POLDIP3* mRNA levels in culture cells transfected with *TDP-43* 3′-UTR siRNA as a fold change relative to those of cells transfected with control siRNA. The amounts of variant-1 (**D**), variant-2 (**E**) or total *POLDIP3* mRNA (**F**) were quantified by real-time qRT-PCR using the primers indicated with arrows in Fig. 2A. In this experiment, *RPLP1* and *RPS18* were used as reference genes. Data represent the mean with standard error from three independent experiments. Asterisk indicates significant difference among *TDP-43* 3′-UTR siRNA-transfected cells (*P<0.01, Tukey multiple comparison test).

To investigate whether TDP-43 directly regulates the inclusion of exon 3 of *POLDIP3*, we first examined whether TDP-43 binds to the *POLDIP3* mRNA by RNA-immunoprecipitation (RIP) assay using TDP-43 antibody. *POLDIP3* mRNA was amplified in the material immunoprecipitated with anti-TDP-43 antibody (Fig. 3A, lane 4, top panel).

Next, we applied a supplementation assay by introducing the exogenous mutant TDP-43 in the cell depleted of endogenous TDP-43 by using siRNA targeted to the 3′-UTR of *TDP-43.* TDP-43 has two RNA recognition motifs (RRM1 and RRM2) and the substitutions of F147L and F149L in the RRM1 motif decreased the binding ability to RNA, but the substitutions of F194L, F229L, and F231L in the RRM2 motif did not (Fig. 3B) [8]. We treated the HeLa cells by siRNA targeted to the 3′-UTR of *TDP-43* then transfected the various TDP-43 cDNAs and quantified the amount of *POLDIP3* variants by real-time qRT-PCR. Although exogenous expression of TDP-43 wild-type and each mutant did not affect the expression of *POLDIP3* total and variant-2 mRNA (Fig. 3E and F), wild-type TDP-43 restored the amount of *POLDIP3* variant-1 (Fig. 3C and D, lane 3). In contrast, TDP-43 RRM1 mutant, but not RRM2 mutant, failed to restore the amount of *POLDIP3* variant-1 (Fig. 3D, lanes 4 and 5). These results suggest that TDP-43 includes exon 3 of *POLDIP3* by binding to its pre-mRNA.

### Alteration of *POLDIP3* splicing in the CNS of patients with ALS

**Figure 4:**
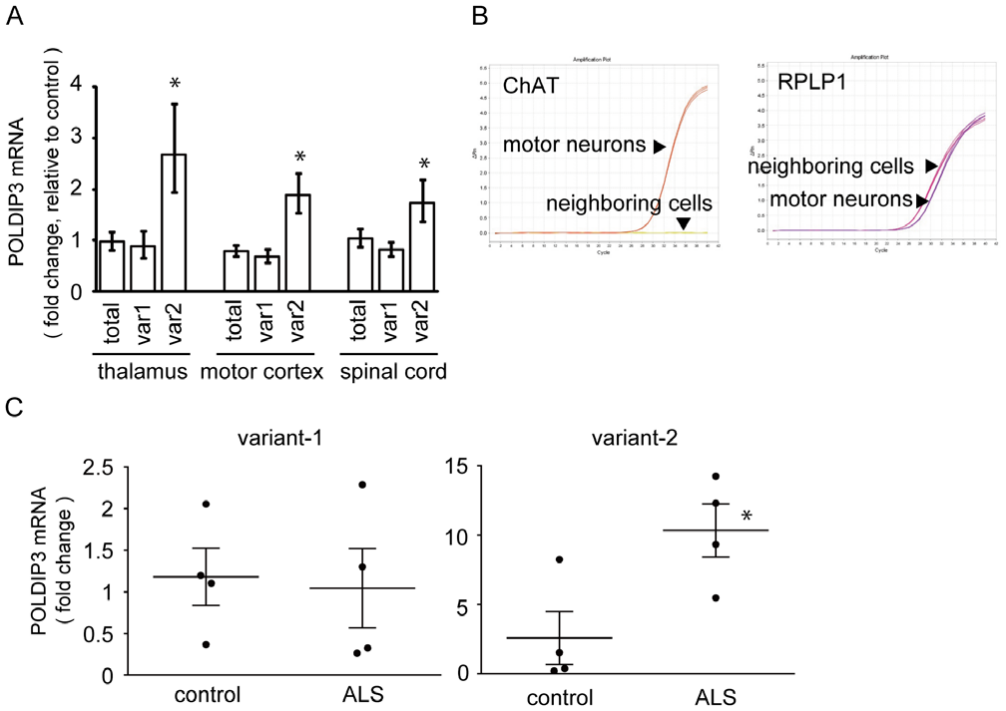
Increased *POLDIP3* variant-2 expression in the CNS with ALS. (**A**) Increased *POLDIP3* variant-2 in CNS with ALS. Total RNA was isolated from the thalamus, motor cortex, and spinal cord of controls (*n* = 5−7) and patients with ALS (*n* = 6−7) and qRT-PCR analysis was performed using the primers indicated with arrows in Fig. 2A. *POLDIP3* mRNA of total, variant-1, and variant-2 are represented as a fold change relative to controls. In this experiment, *RPLP1* and *RPS18* were used as reference genes. Data represent the mean with standard error. Asterisk indicates significant difference (*P<0.05, Student *t* test). (**B**) Purity of the motor neuron enriched material obtained by laser microdissection methods. We isolated 100 motor neurons from L2 in each individual control (*n* = 4) or individual with ALS (*n* = 4). Real-time qRT-PCR revealed that Tthe expression level of *ChAT* mRNA, a motor neuron marker, was only detected in the motor neuron-enriched material. In contrast, similar expression levels of *RPLP1* mRNA, which was one of the internal controls used in this experiment, were detected in both materials. (**C**) Increased *POLDIP3* variant-2 in ALS motor neurons. *POLDIP3* variant-1 and variant-2 mRNA levels as a fold change relative to those of control are presented by a scatter plot. In this experiment, *RPLP1* and *RPS18* were used as reference genes. Solid line represents the mean with standard error. Asterisk indicates significant difference (*P<0.05, Student *t* test).

To investigate the function of TDP-43 in tissues affected with ALS, we analyzed the amount of each *POLDIP3* splicing variant mRNA. We isolated total RNAs from the thalamus, motor cortex and spinal cord of patients with ALS and control individuals (detailed information about subjects is summarized in Table S1) and quantified the amounts of mRNA of *POLDIP3* splicing variants using real-time qRT-PCR. The total amounts of *POLDIP3* and *POLDIP3* variant-1 mRNA were not significantly different between control and ALS tissues (Fig. 4A). In contrast, the amount of *POLDIP3* variant-2 was significantly increased in ALS tissues compared with controls (Fig. 4A). An increase of *POLDIP3* variant-2 mRNA was also observed in the thalamus, which has not been thought to be involved in ALS. However, consistent with the previous report, TDP-43 pathology was observed in four out of six ALS cases used in this experiment [22] (Fig. S2).

Next we investigated the amounts of *POLDIP3* mRNA in the spinal motor neurons of patients with ALS. We isolated at least 100 spinal motor neurons at L2 in each individual with ALS and controls by laser capture microdissection. The extracted RNA was retrotranscribed, pre-amplified, and subsequently analyzed by real-time qRT-PCR. To assess the purity of the motor neuron-enriched material, we quantified the amount of choline acetyltransferase (*ChAT*) mRNA, one of the markers for motor neurons, and compared it to neighboring cells. Similar expression levels of ribosomal protein, large P1 (*RPLP1*), which was one of the internal controls used in this experiment, was detected between them. In contrast, the expression of *ChAT* mRNA was only detected in the motor neuron-enriched material (Fig. 4B). The amount of expression of *POLDIP3* variant-1 in spinal motor neurons was similar between ALS and controls (Fig. 4C). However, the amounts of expression of *POLDIP3* variant-2 in ALS tissues was statistically increased over 4 times compared to controls (Fig. 4C).

### The function of POLDIP 3 variant-2

**Figure 5:**
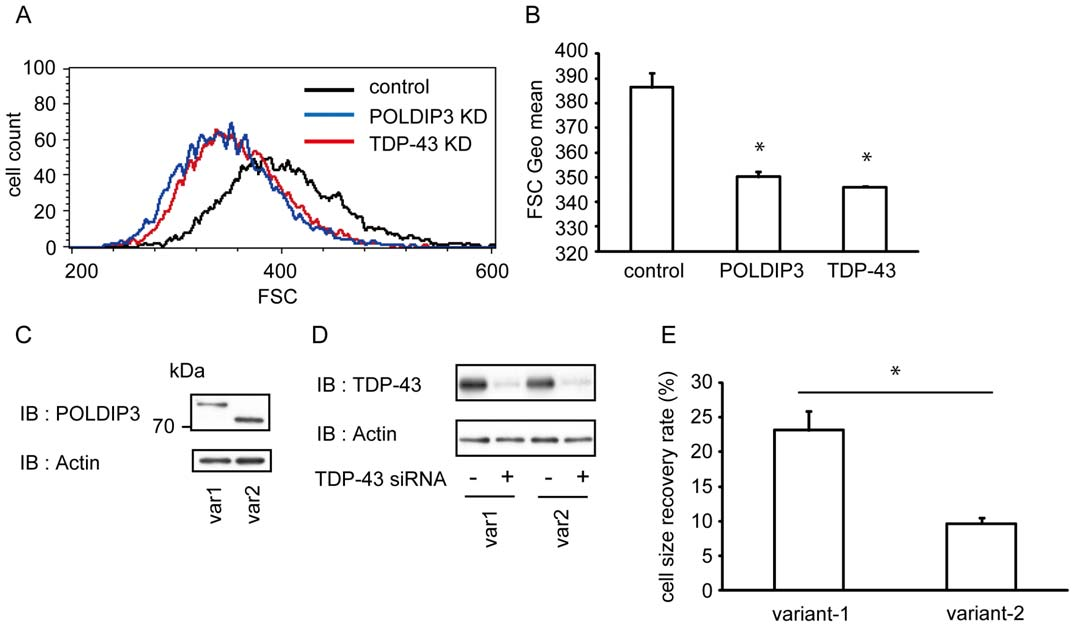
Reduction of TDP-43 expression makes cells smaller. (**A**) A flow cytometer was used to obtain forward scatter (FSC) histograms of SH-SY5Y cells transfected with siRNA targeting POLDIP3 (blue) or TDP-43 (red) or non-targeting control (black). The FSC histogram is one representative transfection from which 10,000 cells were counted. (**B**) Data represent the mean with standard error of forward scatter of the siRNA-transfected cells from three independent experiments. Note that down-regulation of TDP-43 leads to a reduction in cell size as well as POLDIP3 depression. Asterisk indicates significant difference (*P<0.01, Tukey multiple comparison test). (**C and D**) Cell lysates from the SHSY5Y cells stably expressed GFP-tagged POLDIP3 variants were subjected to immunoblotting for POLDIP3 (**C**) or TDP-43 (**D**). Anti-actin immunoblotting served as a loading control. (**E**) Recovery rate of the cell size in the TDP-43-depleted SH-SY5Y with or without expression of GFP-tagged POLDIP3 variant. Data represent the mean with standard error from three independent experiments. Note that the cell size recovery rate is significantly increased in the cells expressing POLDIP3 variant-1 compared to in the cells expressing variant-2. Asterisk indicates significant difference (*P<0.01, Student *t* test).

Finally, we were interested in the function of POLDIP3 variant-2. POLDIP3 has been reported to enhance mammalian target of rapamycin (mTOR)/S6 protein kinase 1 (S6K1)-mediated translation efficiency of mRNA [23]. mTOR/S6K1 signal functions in cell proliferation and growth [24]. Indeed, the depletion of POLDIP3 results in decreased cell size [25]. Therefore, to estimate the function of POLDIP3 variant-2, we investigated the cell size in a neuronal cell line, SH-SY5Y, with or without TDP-43 siRNA. In SH-SY5Y cells treated with TDP-43 siRNA, cell size was significantly decreased and was comparable to depletion of POLDIP3 (Fig. 5A and B) [25]. In contrast, in HeLa and HEK293T cells treated with TDP-43 siRNA, cell size was significantly increased, which is consistent with a previous report (Fig. S3A and B) [26]. In addition, although there was no statistical significance, cell size in U87-MG cells treated with TDP-43 siRNA showed a tendency to be larger (Fig. S3C).

To further elucidate the functional difference between POLDIP3 variant-1 and POLDIP3 variant-2, we investigated whether these POLDIP3 variants can restore the size of SH-SY5Y cells treated with TDP-43 siRNA. We transfected SH-SY5Y cells, which stably expressed the GFP-tagged POLDIP3 variant-1 or variant-2, with control or TDP-43 siRNA. Then we calculated the cell size recovery rate by comparing the cell size between the cells with or without the expression of GFP-tagged POLDIP3 variants (Fig. 5C; Fig. S4). TDP-43 siRNA treatment suppressed the expression of TDP-43 to <20% in both POLDIP3 stable cell lines compared to control siRNA (Fig. 5D). Although both POLDIP3 variants improved the cell size reduction caused by depletion of TDP-43, the recovery rate of cell size was higher in variant-1-expressing cells than in variant-2-expressing cells and reached statistical significance, indicating that the POLDIP3 variant-2 is less effective at maintaining the size of SH-SY5Y cells than variant-1 (Fig. 5E).

## Discussion

Here, we show the increasing the amounts of the *POLDIP3* variant-2 mRNA in the affected tissues with ALS, including spinal motor neuron. The result suggests that a function of TDP-43 decreases in the affected tissues with ALS. The finding that *POLDIP3* variant-2 increased in the cultured human cells depleted of TDP-43 is consistent with the previous reports [19], [20]. In addition, we and Fiesel *et al.* found that RNA binding ability of TDP-43 is necessary for inclusion of exon 3 of *POLDIP3*, indicating that TDP-43 directly regulates splicing of *POLDIP3* [26]. Therefore we conclude that the alteration of splicing of *POLDIP3* represents the dysfunction of TDP-43. The finding that the TDP-43 function decreases in the affected tissue with ALS supports the hypothesis that a loss of function of TDP-43 underlies the pathogenesis of ALS.

We were not able to show the decreasing the amounts of *POLDIP3* variant-1 in the affected tissues with ALS. There is a possibility that the increasing the amount of *POLDIP3* variant-2 might be resulted from the mechanism which is not associated with the TDP-43 function. However, the reason why the decreasing the amounts of *POLDIP3* variant-1 was not observed in affected tissues with ALS is related to the number of the neurons and glial cells in which *POLDIP3* splicing altered. The alteration of *POLDIP3* splicing may occur only in the cells with disappearance of nuclear TDP-43. The number of these cells is relatively small even though in the spinal motor neurons in ALS [27]. In addition, the amount of *POLDIP3* variant-1 is abundant in normal cells, thus it would be difficult to detect the decreasing the amounts of *POLDIP3* variant-1. In contrast, the *POLDIP3* variant-2 is nearly absent in normal cells (Fig. 2B), thus increasing the amounts of *POLDIP3* variant-2 is detected sensitively. The immunohistochemical analysis to compare the amount of variant-1 between the cells with or without nuclear TDP-43 should clarify this issue; however, the POLDIP3 antibodies evaluated in this study were not suitable for immunohistochemical analysis in human autopsied tissues (data not shown). Further study by using the specific antibodies for POLDIP3 variant-1 is necessary to elucidate this issue.

TDP-43 functions in exon inclusion as well as exon exclusion [19], [20]; however, the precise mechanism for regulation of splicing by TDP-43 is unclear. TDP-43 binds to the intronic or noncoding sequences, preferentially (UG)n sequence, and the position of the TDP-43 binding site is associated with exclusion or inclusion of an exon [19], [20]. We have shown that only a small number of the genes, which alter their splicing by depletion of TDP-43, are overlapped between HeLa and SH-SY5Y cells. The result indicates that the splicing regulation of TDP-43 is not simply resulted from the nucleotide sequence but also may be regulated by the interaction with other splice factors, including hnRNP A1, A2, and A2/B1 in each cell [10], [11].

Although several studies have reported that the pre-mRNA splicing of genes, including *CFTR*, *apoA-II* and *SMN2*, was regulated by TDP-43 in vitro [8], [28], [29], our results show that depletion of TDP-43 does not affect the splicing for these genes. However, depletion of TDP-43 altered the pre-mRNA splicing in many genes as previously reported [19], [20]. In contrast, relatively small numbers of genes were significantly altered in their amount of expression by depletion of TDP-43. Our results indicated that TDP-43 has relatively small effects in the regulation of the amounts of mRNA than the regulation of the splicing in culture cells.

A recent study revealed the alteration of splicing in the frontal cortex with frontotemporal lobar degeneration with TDP-43-positive inclusions (FTLD-TDP), which is another type of TDP-43 proteinopathy [30]. They found an alteration of splicing for *BIM* (Bcl-2 interacting mediator of cell death) but not for *POLDIP3* in brain from FTLD-TDP. The reason why the alteration of splicing for *POLDIP3* is not observed in their analysis might be related to the difference of analysed region, frontal cortex and motor cortex or spinal cord, or the difference of the residual TDP-43 function. Indeed the intracellular and intra central nervous system distribution of TDP-43-positive inclusions are different between FTLD-TDP and ALS [31], [32].

POLDIP3 is a substrate for S6K1, which is downstream of mTOR, and enhances the mTOR/S6K1-dependent translation of mRNA, which is associated with cell size [23], [25]. Although it has been shown that the POLDIP3 variant-2 locates in the nucleus as well as variant-1 and retains the functional motif, the function of POLDIP3 variant-2 has not been well elucidated [21], [25]. Most recently, it has been reported that the POLDIP3 variant-2 enhanced the mTOR/S6K1-dependent translation of mRNA more, and the depletion of TDP-43 increased cell size in HEK293E cells [26]. The results of our cell size assay in non-neuronal cell lines were consistent with this report. However, we have shown that the depletion of TDP-43 decreased cell size in the SH-SY5Y neuronal cell line. The difference for the effect of the depletion of TDP-43 on cell size could be explained by the cell type difference, neuronal or non-neuronal cell line. In addition, by the rescue experiment for cell size, we showed that POLDIP3 variant-2 is less effective than POLDIP3 variant-1 in regard to improving the size of SH-SY5Y cells reduced by the depletion of TDP-43 (Fig. 5E). Thus, the loss of function of TDP-43 may induce decreased function of POLDIP3 due to alteration of *POLDIP3* splicing, which could result in down-regulation of mTOR/S6K1-dependent translation of mRNA in the neuronal cell line. Although loss of TDP-43 function affects the expression and the splicing of various genes, it would be interesting to investigate the contribution of the alteration of *POLDIP3* splicing to the pathogenesis of ALS.

In conclusion, we have shown that the amounts of *POLDIP3* variant-2 mRNA increased in affected tissues with ALS, indicating the loss of function of TDP-43 in ALS. In addition, increasing the amount of *POLDIP3* variant-2 has a potential to be an ideal and sensitive biomarker to represent the dysfunction of TDP-43, since the *POLDIP3* variant-2 is rarely expressed in normal tissues. Thus, the development of the method to detect the amounts of POLDIP3 variant-2 in cerebrospinal fluid would be interesting to diagnosis and evaluate the progression of ALS.

## Materials and Methods

### Subjects

Details of the patients and controls included in this study are compiled in Table S1. This study was approved by the Institutional Review Board of Niigata University, and written informed consent was obtained from families.

### Plasmid constructs

Full-length human TDP-43 complementary DNA (cDNA) and full-length human POLDIP3 cDNA were isolated from the human whole-brain cDNA library (Clontech, Palo Alto, CA, USA) and subcloned into the pcDNA DEST-40 (Invitrogen, Carlsbad, CA, USA) and Vivid colors pcDNA6.2/EmGFP vector (Invitrogen). Human POLDIP3 variant-2 expression plasmid was generated using KOD plus mutagenesis kit (TOYOBO, Osaka, Japan). Mutant TDP-43s (mRRM1, mRRM2, mRRM1/2) expression plasmids were generated with the use of the GeneTailor site-directed mutagenesis system (Invitrogen).

### Cell culture and transfection

We cultured HeLa, SH-SY5Y, U87-MG, and HEK293T cells (American Type Culture Collection, Manassas, VA, USA) in Dulbecco modified Eagle medium supplemented with 10% fetal bovine serum at 37°C in 5% CO_2_. Plasmid DNAs were transfected into these cells with Lipofectamine 2000 (Invitrogen) according to the manufacturer's instructions. siRNA transfections were performed twice using lipofectamine RNAi MAX (Invitrogen) with 100 pmol control (ID: D-001810-10) or TDP-43-specific (L-012394-00) or POLDIP3-specific (L-019036-01) siRNAs in 6-well plates (Thermo Fisher Scientific, Bremen, Germany). For a selective depletion for endogenous TDP-43, but not for exogenous TDP-43, we used siRNA targeted to the 3′-UTR of TDP-43 (Hokkaido System Science, Sapporo, Japan). The sequence of TDP-43 siRNA for 3′-UTR as follows: 5′-GAGACUUGGUGGUGCAUAA-3′.

### Western blot analysis

Cells were lysed in RIPA buffer (25 mM Tris-HCl pH 7.6, 150 mM NaCl, 1% NP-40, 1% sodium deoxycholate, 0.1% sodium dodecyl sulfate [SDS]) with protease inhibitor cocktail (1∶200, SIGMA, St. Louis, MO, USA) at 4°C. Lysates were centrifuged for 10 minutes at 10,000 *g*, and the resulting supernatants were collected. Protein concentration was determined by BCA protein assay kit (Pierce, Rockford, IL, USA), and equal amounts of protein from cell lysates were analyzed. Lysates were subjected to SDS-polyacrylamide gel electrophoresis and transferred onto polyvinylidene difluoride membranes (Millipore, Bedford, MA, USA). Membranes were incubated with primary antibodies overnight at 4°C, followed by horseradish peroxidase (HRP)-conjugated secondary antibody (1∶10,000, SIGMA). TDP-43 was detected with anti-TDP-43 antibody (1∶5000, ProteinTech Group, Chicago, IL, USA). POLDIP3 variant-1 was detected with anti-POLDIP3 antibody (1∶1000; Cell Signaling, Beverly, MA, USA), and POLDIP3 variant-2 was detected with another anti-POLDIP3 antibody (1∶200; Santa Cruz Biotechnology, Santa Cruz, CA, USA). To detect both POLDIP3 variants in Fig. 5C, we used monoclonal rabbit anti-POLDIP3 antibody (1∶1000, cell signaling). We detected actin as a loading control using anti-actin antibody (1∶1000; Santa Cruz Biotechnology).

### Exon array analysis

The Affymetrix GeneChip Whole Transcript Sense Target Labeling Assay was used to generate amplified and biotinylated sense-strand DNA targets (Affymetrix, Santa Clara, CA, USA). Probe was hybridized to Affymetrix 1.0 Human Exon ST arrays according to the manufacturer's instructions (Affymetrix). This array is able to determine the difference at both exon- and gene-level expressions. The arrays were scanned with an Affymetrix Gene Chip scanner 3000 system (Affymetrix). Initial data were processed to CEL file using GeneChip Operating Software (Affymetrix). Analysis of microarray data was performed with Genespring GX ver.11 (Agilent Technologies, Palo Alto, CA, USA). CEL files were imported into the software and their backgrounds underwent Robust Multichip Average and quantile normalizations. For our analysis, we have used only the core probe set. Statistical analysis was performed by creating two groups from the individual CEL files, transfection with control siRNA or with TDP-43 siRNA, and then comparing them. From these array data, we calculated the *P* value the splicing index (SI), and fold change. Both a *P*<0.05 and an |SI|>0.5 were used as thresholds to identify the aberrant spliced genes, and both a *P*<0.05 and a fold change 2.0 were used for determining differentially expressed genes.

### Real-time quantitative RT-PCR analysis

Total RNA was isolated from siRNA transfected cells with the RNeasy plus mini-kit (Qiagen, Valencia, CA, USA). cDNA was synthesized with a high-capacity cDNA reverse transcription kit (Applied Biosystems, Foster City, CA, USA). For autopsied human frozen tissues, mirVana miRNA isolation kit was used to isolate total RNA (Applied Biosystems), followed by synthesis of cDNA using SuperScript® VILOTM cDNA Synthesis Kit (Invitrogen). The RNA quality was assessed by the RNA integrity number (RIN) of the Agilent Bioanalyzer 2100 (Agilent Technologies). RIN score in each sample were described in Table S1. Real-time qRT-PCR was conducted with SYBR Premix Ex Taq on the Thermal Cycler Dice (Takara, Shiga, Japan). Thermal cycler setting was 1) 95°C, 30 sec, 2) 95°C, 15 sec, 3) 60°C, 30 sec and repeat 40 cycles. In each qRT-PCR experiments, Ribosomal protein, large P1 (*RPLP1*) and ribosomal protein S18 (*RPS18*) were used as reference genes, which are determined by geNorm [33]. We designed primer pairs for *POLDIP3* variant-1 and *POLDIP3* variant-2 as follows: POLDIP3 variant-1F: 5′-CAAAACCATCCAGGTTCCAC-3′, POLDIP3 variant-1R: 5′-AAATTCTGTTTGGCCTGGTG-3′, POLDIP3 variant-2F: 5′- GCTCACCAAAACCATCCAGAA-3′, POLDIP3 variant-2R: 5′- ACTGCTTAGCCCAGCCATGT-3′. The primer design for *MMAB*, *HP1BP3*, *GLS*, *STRA6* and DPM2 as follows: MMAB whole-F: 5′- CAGTGCACATTGCAGGACGTC -3′, MMAB whole-R: 5′- GTACTTGTCGATCCACTGCTC -3′, MMAB variant-F: 5′- CTTAACACTTACCTGTTCCTG -3′, MMAB variant-R: 5′- GAAGCTCTTCGGCAAATGTATG -3′, HP1BP3 whole-F: 5′- GTGAACTCGTCCATCCTAAGG -3′, HP1BP3 whole-R: 5′- CACAGTTCGACGAATCGGCATG -3′, HP1BP3 variant-F: 5′- CTACTTCGAGTGAGGCAGAGC -3′, HP1BP3 variant-R: 5′- GAGTGGAATGAATACTATCAGCTC -3′, GLS whole-F: 5′- GAGTACTGAGCCCTGAAGCAG -3′, GLS whole-R: 5′- CCAGATTTTGCAGGAAGACCAAC -3′, GLS variant-F: 5′- CTTGATCCTCGAAGAGAAGG -3′, GLS variant-R: 5′- CTCATTTGACTCAGGTGACAC -3′, STRA6 whole-F: 5′- GACTACTCCTATGGCAGCTG -3′, STRA6 whole-R: 5′- CACACAGTCAGGCCAGAGCTG -3′, STRA6 variant-F: 5′- CTCCTGAGCTCCCTGTGTTTG -3′, STRA6 variant-R: 5′- GACTAGTCTCAACTCTGTGATC -3′, DPM2 whole-F: 5′- GTCGCCGTTAGCCTGATCATC -3′, DPM2 whole-R: 5′- CAGGAAATACTTGTGGATGAC -3′, DPM2 variant-F: 5′- CAGCATGTCATCCACAAG -3′, DPM2 variant-R: 5′- CTCTTGGTCTTCAGCATCAC -3′. The specificity of each primer was examined using the clones that contained each splicing variant of *POLDIP3*. Predesigned primers were used for *POLDIP3* total, *RPLP1*, and *RPS18* (Takara).

### RNA immunoprecipitation (RNA-IP)

RNA-IP was performed using the Magna RIP RNA-Binding Protein Immunoprecipitation Kit following the manufacturer's instructions (Millipore, Billerica, MA, USA). Endogenous TDP-43 protein was immunoprecipitated with anti-TDP-43 antibody (ProteinTech Group) from HeLa cell lysates, and the RNAs isolated from the immunoprecipitated material were subjected to RT-PCR for the *POLDIP3* transcripts. Control rabbit IgG was used as a negative control (Abcam, Cambridge, MA, USA). The primer pairs for *POLDIP3* are as follows; POLDIP3 RNA-IP F: 5′-GCTAAGCAGTTCCAAGCTTTCC-3′, POLDIP3 RNA-IP R: 5′-TGGCAGCTCTTTGGGGGGTT-3′.

### Microdissection, total RNA isolation, and qRT-PCR analysis

Frozen sections of spinal cord (10 µm) were stained with HistoGene staining kit (Arcturus Bioscience, Mountain View, CA, USA), followed by microdissection on AS LMD Laser Capture Microdissection System (Leica, Wetzlar, Germany). RNA isolation from microdissected cells was performed using RNAqueous micro kit (Ambion, Austin, TX, USA). cDNA was synthesized with a high-capacity cDNA reverse transcription kit (Applied Biosystems). Before qRT-PCR analysis, cDNA was pre-amplified using the *Taq*Man PreAmp Master Mix (Applied Biosystems) according to the manufacturer's instructions. *Taq*Man gene expression assays were used to determine the expression of *POLDIP3* variant-1 (assay ID: Hs00993978_m1), *POLDIP3* variant-2 (Hs00994038_m1), *RPLP1* (Hs01653088_g1), *RPS18* (Hs02387368_g1), and *ChAT* (Hs00252848_m1) (Applied Biosystems).

### Immunohistochemical analysis

Cells were fixed with 4% paraformaldehyde, permeabilized in 0.2% Triton X-100 for 15 minutes and then labeled with primary antibodies. The following antibodies were used: anti-TDP-43 antibody (1∶200, Abnova, Taipei, Taiwan), anti-POLDIP3 variant-1 antibody (1∶100, Cell Signaling). Images were obtained with an inverted microscope (TE-300NT; Nikon, Tokyo, Japan) and a confocal microscope (CSU-10; Yokogawa Electric, Tokyo, Japan) equipped with a ×100 objective (NA 0.80; Olympus, Tokyo, Japan).

### Cell size assay

For determining the cells in G_1_ phase, we stained the cells with 7-amino-actinomycin D (7-AAD) according to the manufacturer's instructions (BD Biosciences, San Jose, CA). Briefly, cells were fixed and permeabilized with ice cold 80% methanol and incubated for 1 minute on ice. After washing twice with phosphate-buffered saline, cells were incubated with 7-AAD (SIGMA) for 15 minutes at room temperature in the dark. DNA content was determined by FACSCalibur^TM^ (BD Biosciences) and data were analyzed using CellQuest Pro software (BD Biosciences). The cells with G_0_/G_1_ phase DNA content were analyzed to determine the cell size.

To further elucidate the functional difference between POLDIP3 variant-1 and POLDIP3 variant-2, we attempted to investigate cell size following treatment with TDP-43 siRNA in SH-SY5Y cells that stably expresses GFP-tagged POLDIP3 variant-1 or variant-2. To estimate the recovery rate of cell size correctly, we used GFP-negative cells in each population as controls. We transfected the cells with TDP-43 or control siRNAs, followed by measuring cell size in each population by FACSCalibur^TM^ (BD Biosciences) and analyzing data by using CellQuest Pro software (BD Biosciences). From the measured value of cell size, we calculated the recovery rate of cell size by comparing the measured value between the cells with or without expression of GFP-tagged POLDIP3 variant. The equation for calculating the recovery rate is as follows:



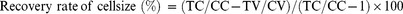



TC  =  forward scatter of control cells treated with TDP-43 siRNA, CC  =  forward scatter of control cells treated with control siRNA, TV  =  forward scatter of cells expressed GFP-tagged POLDIP3 variant treated with TDP-43 siRNA, and CV  =  forward scatter of cells expressed GFP-tagged POLDIP3 variant treated with control siRNA. The schematic diagram of the cell size rescue experiment is also shown in Fig. S4.

## Supporting Information

Figure S1 **qRT-PCR analysis for validation of exon array in other 4 genes.** (**A**) Exon structure diagram of the main isoform (top) and candidate variant (bottom) for *MMAB, GLS, STRA6, DPM2.* From the exon array result, we selected the candidate splicing variant that was induced by the depletion of TDP-43. The name of each transcript is labeled on the side of the exon structure. The red box indicates the exons that are expected to be altered by TDP-43 depletion. The black bars indicate the position of the primers we used in this experiment. The gene views show the expression of exons as determined by analyzing the results of exon array in HeLa cells using Genespring GX. The expression levels are shown on a log2 scale; the error bars show standard errors of means. TDP-43 siRNA, red circle; control siRNA, blue square. (**B**) qRT-PCR analysis revealed that the splicing alteration of these genes was not validated. *RPLP1* and *RPS18* were used as reference genes. Data represent the mean with standard error from three independent experiments. Asterisk indicates significant difference (*P<0.01, Student *t* test).
(TIF)

Figure S2 **Immunohistochemical analysis for TDP-43 in the thalamus.** To investigate the presence of TDP-43 pathology in the thalamus from ALS cases used in Figure 4A, TDP-43 immunostaining was performed. Four out of six ALS cases showed (**A**) glial, and (**B–D**) neuronal cytoplasmic TDP-43 immunoreactivity. The label on each figure corresponds to the ID described in Table S1. Scale bar, 20 µm.
(TIF)

Figure S3 **Cell size analysis for TDP-43 depressed non-neuronal cells.** Data represent the mean with standard error of forward scatter of control or TDP-43 siRNA-transfected cells from three independent experiments. Note that down-regulation of TDP-43 leads to increase cell size in HeLa and HEK293T cells. Asterisk indicates significant difference (*P<0.01, Student *t* test).
(TIF)

Figure S4 **The schematic diagram for the cell size rescue experiment by expression of exogenous POLDIP3 variants.**
(TIF)

Table S1 **Clinical and RNA data for samples in this study.** The detailed data for each sample of human material used in this study is shown. Circle indicates the usage for the experients labeled on the top of column. No individual with ALS has a mutation in the *TARDBP* gene. PMI: Post Morten Interval, RIN: RNA Integrity Number.
(XLS)
