# Supplementary figures and images for: Alteration of POLDIP3 Splicing Associated with Loss of Function of TDP-43 in Tissues Affected with ALS
Source: PLoS One. 2012 Aug 10;7(8):e43120. doi: 10.1371/journal.pone.0043120 (PMC3416794; doi:10.1371/journal.pone.0043120)

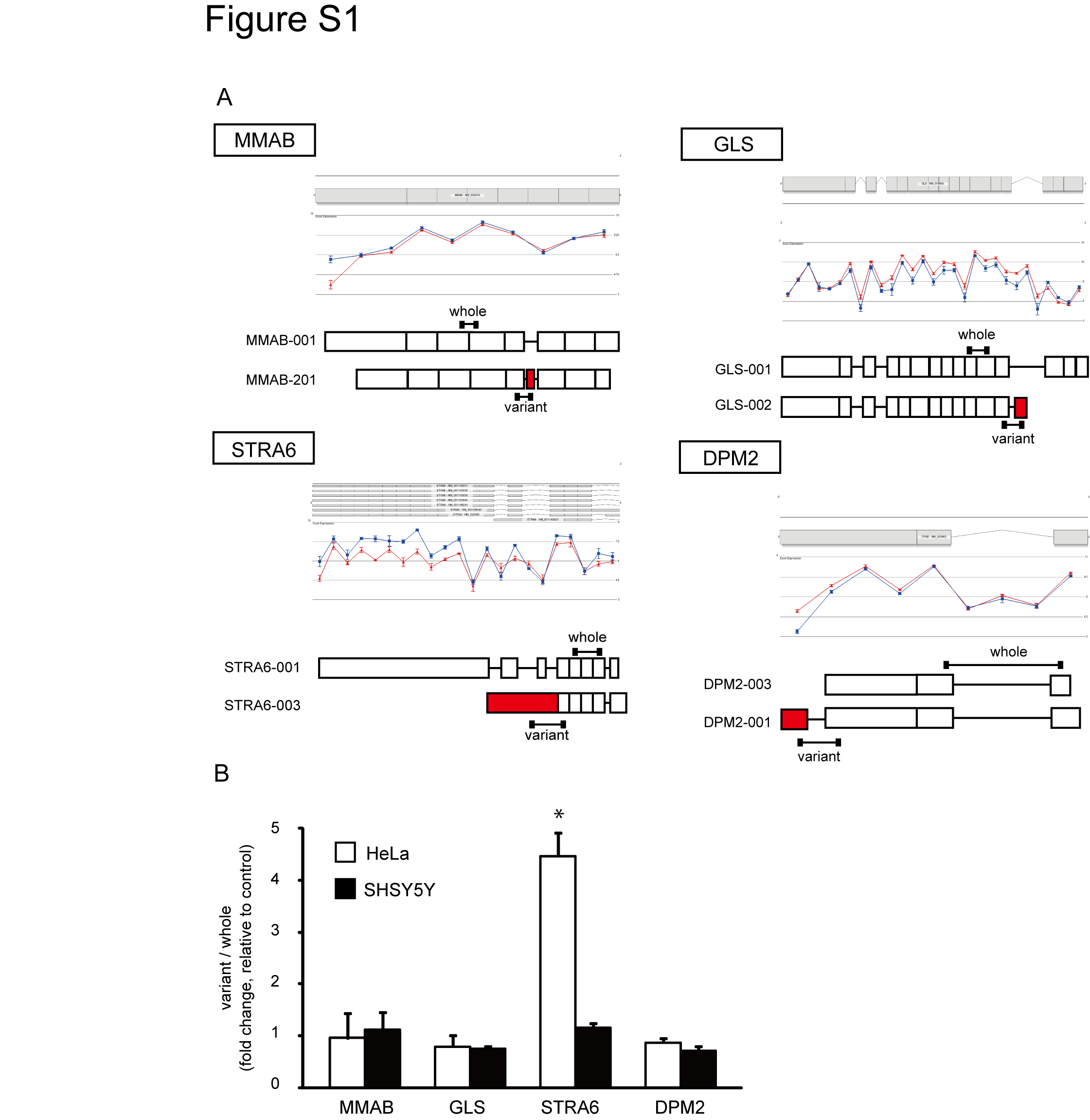

Supplement: Figure S1 — qRT-PCR analysis for validation of exon array in other 4 genes. (A) Exon structure diagram of the main isoform (top) and candidate variant (bottom) for MMAB, GLS, STRA6, DPM2. From the exon array result, we selected the candidate splicing variant that was induced by the depletion of TDP-43. The name of each transcript is labeled on the side of the exon structure. The red box indicates the exons that are expected to be altered by TDP-43 depletion. The black bars indicate the position of the primers we used in this experiment. The gene views show the expression of exons as determined by analyzing the results of exon array in HeLa cells using Genespring GX. The expression levels are shown on a log2 scale; the error bars show standard errors of means. TDP-43 siRNA, red circle; control siRNA, blue square. (B) qRT-PCR analysis revealed that the splicing alteration of these genes was not validated. RPLP1 and RPS18 were used as reference genes. Data represent the mean with standard error from three independent experiments. Asterisk indicates significant difference (*P<0.01, Student t test). (TIF) [file pone.0043120.s001.tif]

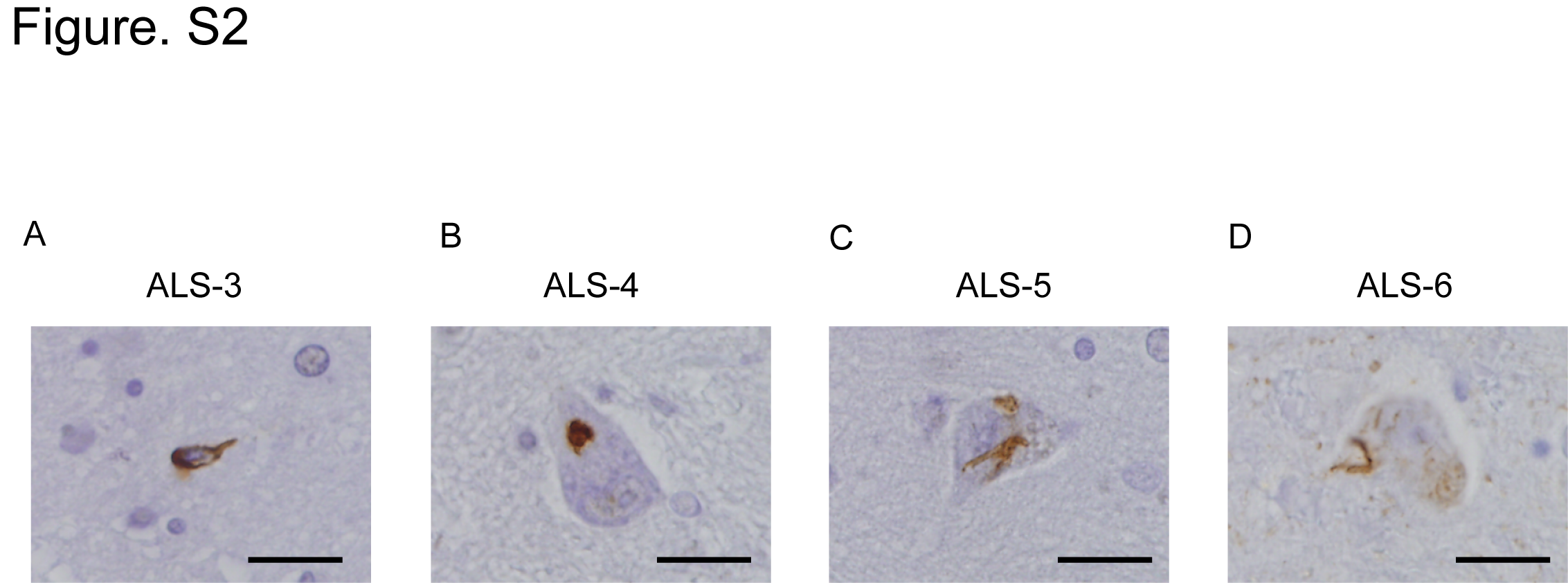

Supplement: Figure S2 — Immunohistochemical analysis for TDP-43 in the thalamus. To investigate the presence of TDP-43 pathology in the thalamus from ALS cases used in Figure 4A, TDP-43 immunostaining was performed. Four out of six ALS cases showed (A) glial, and (B–D) neuronal cytoplasmic TDP-43 immunoreactivity. The label on each figure corresponds to the ID described in Table S1. Scale bar, 20 µm. (TIF) [file pone.0043120.s002.tif]

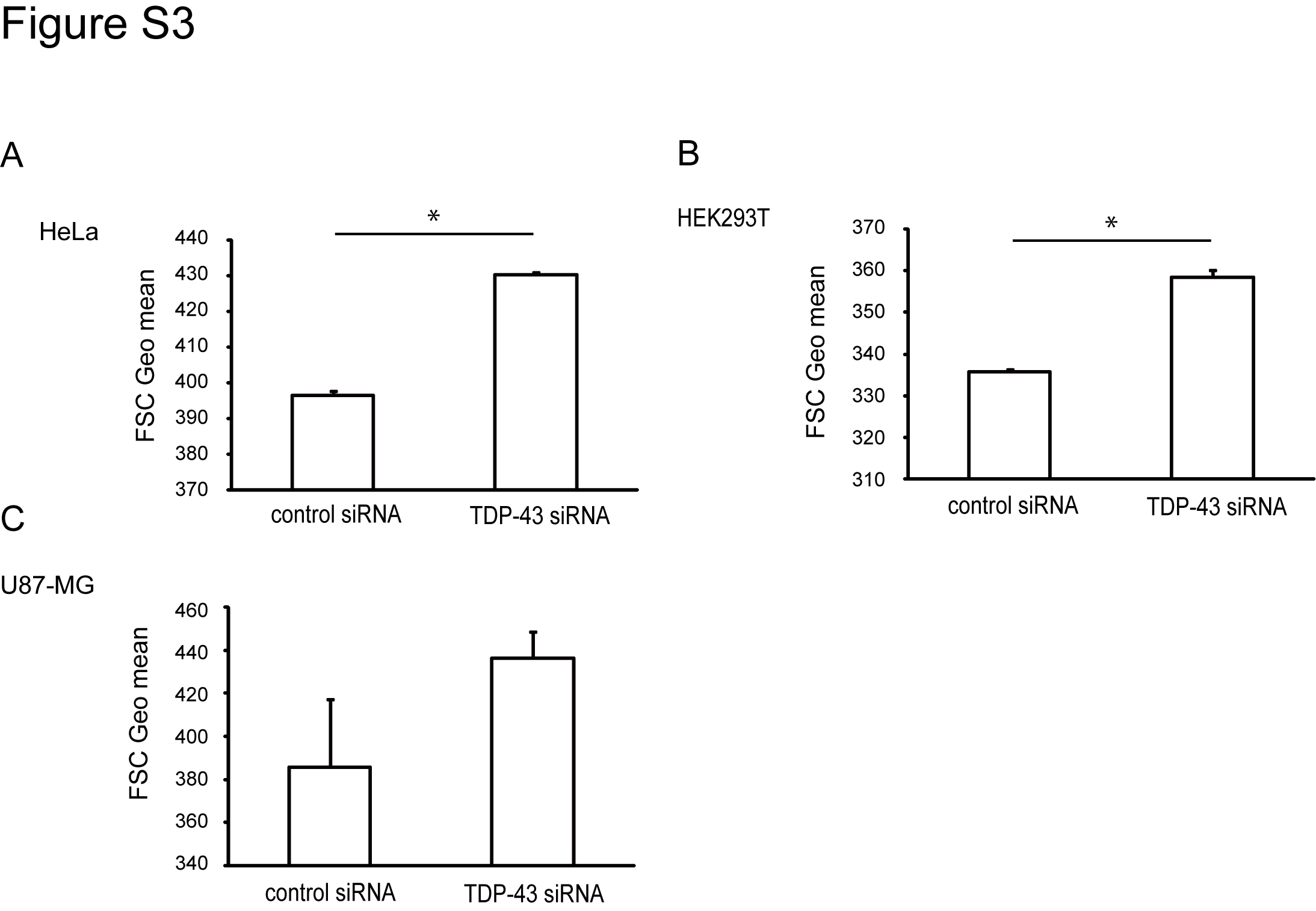

Supplement: Figure S3 — Cell size analysis for TDP-43 depressed non-neuronal cells. Data represent the mean with standard error of forward scatter of control or TDP-43 siRNA-transfected cells from three independent experiments. Note that down-regulation of TDP-43 leads to increase cell size in HeLa and HEK293T cells. Asterisk indicates significant difference (*P<0.01, Student t test). (TIF) [file pone.0043120.s003.tif]

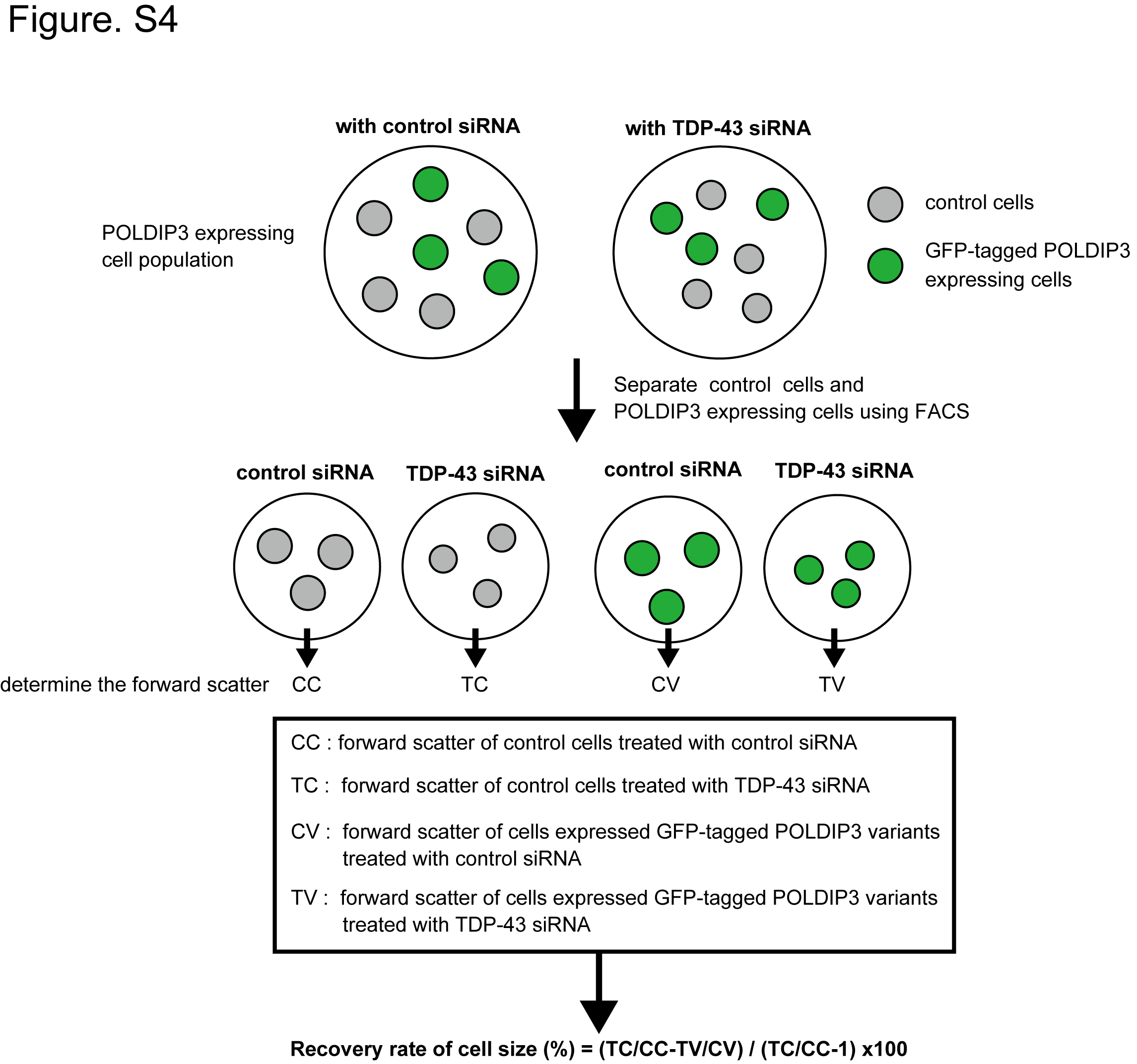

Supplement: Figure S4 — The schematic diagram for the cell size rescue experiment by expression of exogenous POLDIP3 variants. (TIF) [file pone.0043120.s004.tif]
